# News Items

**Published:** 1869-09-15

**Authors:** 


### NEWS ITEMS.

Cheap Enough.—We are enabled to inform the solicitous that an agency has been established in this city for the sale of Philadelphia Diplomas of M.D. Several somewhat notorious characters here have already availed themselves of this royal (albeit, *Paine* ful), road to knowledge. Did not the phrase savor of slang, we might say that the thing is being done *Brown.* We are informed, through a rejoicing M.D., whose parchment was thus procured, that no attendance upon lectures or examinations by the Faculty is required, but simply a certificate from the local agent that the candidate is worthy. So far has this been reduced to a system that the "Dean" of the Faculty actually writes diplomas down (in a note before us) in the same category with post-office orders.

Practical Suggestion.—A valued subscriber writes: "Allow a suggestion in regard to your Reports of Medical Societies. We do not wish to find fault, but, we think they might be made much more interesting to the general reader, if, instead of telling us that A., B. and C. read interesting papers on gun-shot wounds, contagious diseases, typhoid fever, etc.; and Drs. So and So are to investigate certain very important subjects, and report at the next meeting of the Society, they would tell us more of what was said, what old theories were advocated, what new principles were affirmed, what new discoveries made, etc.

In this way we would know what advances they are making in medical science; what efforts they are making for the "elevation" of the profession.

Very respectfully,

T. A. E.

[Which ideas the Editors cordially endorse.]

The San Francisco *Bulletin* says: "It is a pleasure to note that within a few weeks past a substantial progress has been made toward putting the University of California into practical operation. Seven professors, in all, have been elected, viz.: John Le Conte, M.D., Acting President, and Professor of Physics and Industrial Mechanics; Robert A. Fisher, A.M., Professor of Chemistry, Mining and Metallurgy; Joseph Conte, M.D., Professor of Geology, Natural History and Botany; Martin Kellogg, A. M., Professor of Ancient Languages; Paul Pioda, Professor of Modern Languages; Ezra S. Carr, M.D., Professor of Agriculture, Agricultural Chemistry and Horticulture."

The University of California is especially to be congratulated on the appointment of Prof. Carr. Whilst we regret that any concurrence of circumstances removes him so far from our immediate association, we congratulate the new institution on the zeal and ability he will take to it. No better instructor graces the chair of any college on the continent. The best wishes of all who know him will attend him, wherever he goes. Personally, we fervently trust that when the University achieves the great success this appointment almost necessitates, there will not a new king arise who will not know Joseph [Ezra]. This ignorance is often disastrous.

THE COMMITTEE on the Library of the American Medical Association send us the following Circular:

WASHINGTON, D. C.

The Medical Profession, and Scholars generally, are aware of the ephemeral form in which most of the early American contributions to the literature of medicine were given to the world, and, indeed, in which many of the more recent are being published. This condition of much of our professional literature is deeply regretted by all, and particularly by those whose taste and research lead them to refer to this class of works, when the fact is made apparent that whole editions of tracts and books have entirely perished through neglect. With a view to provide against such a contingency, and preserve, for the benefit of the profession, in some accessible and central locality, copies of all home medical publications, the American Medical Association, at its annual meeting in May last, resolved to establish at Washington, D. C., a Library or Repository of medical works, to which it is believed all the current medical literature of our country will be cheerfully, promptly and constantly contributed.

It is designed that this repository shall contain copies of every contribution by American physicians to the literature and science of medicine, from the earliest settlemeut of our country, no matter how or where published, including all the books, pamphlets, journals, and even unpublished manuscripts that can be collected.

Nearly all physicians have some book or pamphlet of the character indicated, which may contain facts relative to the diseases of his section published nowhere else, which they can contribute without inconvenience, and which of itself is of trifling value, yet when many such treatises are assembled together from all parts of our country, embracing its nosology from the earliest period of its settlement, they will form a collection of the greatest importance to the profession.

The Librarian of Congress has kindly consented to receive and preserve as a special deposit, in the Government fire-proof-building, any collection of medical works the American Medical Association may make, and will catalogue and keep them in condition to be readily consulted. The accommodation thus offered the Association for accumulating and preserving its library free of cost is most generous and most encouraging. Gentlemen having scarce and valuable American medical pnblications will not hesitate to deposit them in sueh a safe, central, and national repository, where they will be preserved from destruction and their usefulness secured to the profession.

An appeal for contributions to this library is now made, personally and distinctly, to each and every American Physician, Medical Publisher, and Editor, to deposit copies of their works in this repository, where they will be carefully kept for reference aud catalogued with the name of the donor.

We, the undersigned, members of the American Medical Association, having been selected to carry into effect, as far as practicable, the resolution of the Association to establish a Library, have now completed all the necessary arrangements for the reception and preservation of those books which may be sent to our care. Contributions of the class of works mentioned, are, therefore, respectfully and earnestly solicited from every source. Packages may be sent by mail or by Adams' express, to either of us, which will be promptly acknowledged on reception, and a record of titles kept. The library-mark of the Association will be pasted on the inside of the cover of each volume, which will contain also the name of the donor.

Hoping that you may further the project to the extent of adding at least your own productions,

We remain, respectfully,

ROBERT REYBURN, M.D., *Librarian.*
JOSEPH M. TONER, M.D., *Library Committee.*

THE LITTLE CORPORAL, the brilliant Western Juvenile (which claims to have a larger circulation than any other magazine), announces that it will come free for October, November and December of this year to all new subscribers for the new year, whose names and money are sent to the publishers before the last of October. Beautiful premiums are offered for clubs. Now is a good time to begin. Price One Dollar a year; sample copy, 12 cents. Address ALFRED L. SEWELL & Co., Publishers, Chicago, Illinois.

THE NEW YORK MEDICAL COLLEGE FOR WOMEN will begin its sixth Annual Term of twenty weeks, at their new College in Twelfth Street, corner of Second Avenue, the first Monday iu November. For announcements, giving full particulars, address, with stamps, the Dean, Mrs. C. S. LOZIER, M.D., or the Secretary, Mrs. C. F. WELLS, Box 730 New York.

